# Association of Renal Resistive Index, Renal Pulsatility Index, Systemic Hypertension, and Albuminuria with Survival in Dogs with Pituitary-Dependent Hyperadrenocorticism

**DOI:** 10.1155/2016/3814034

**Published:** 2016-05-31

**Authors:** Hung-Yin Chen, Yu-Hsin Lien, Hui-Pi Huang

**Affiliations:** ^1^Institute of Veterinary Clinical Science, Veterinary School, National Taiwan University, No. 1, Section 4, Roosevelt Road, Taipei 100, Taiwan; ^2^Azu Clinic for Animals, No. 92, Section 1, Kin-Shan South Road, Taipei 100, Taiwan

## Abstract

An increased renal resistive index (RI) and albuminuria are markers of target organ damage secondary to systemic hypertension. This study evaluated associations between systemic blood pressure (SBP), renal RI, pulsatility index (PI), and albuminuria in dogs with pituitary-dependent hyperadrenocorticism (PDH). Predictors of overall mortality were investigated. Twenty client-owned dogs with PDH and 20 clinically healthy client-owned dogs as matched controls were included. Incidence rates of systemic hypertension (SBP ≥ 160 mmHg), albuminuria, and increased renal RI (≥ 0.70) and PI (≥ 1.45) in the control group were 5%, 0%, 5%, and 0%, respectively, compared to 35%, 40%, 50%, and 35%, respectively, in the PDH group (*P* = 0.001, *P* < 0.001, *P* < 0.001, and *P* = 0.001, resp.). No association between systemic hypertension, renal RI, renal PI, and albuminuria was observed. PDH was the only predictor of albuminuria and increased renal RI. Survival was not affected by increased renal PI, systemic hypertension, or albuminuria. Increased renal RI (≥ 0.70) was the only predictor of overall mortality in dogs with PDH.

## 1. Introduction

Glucocorticoid-induced hypertension is common in human patients with hyperadrenocorticism (HAC). In human patients, systemic hypertension may result from increased cardiac output, total peripheral resistance, and renal vascular resistance [1]. Systemic hypertension leads to increased capillary hydrostatic pressure and hyperfiltration, which may eventually cause glomerulosclerosis and proteinuria [2]. These hemodynamic abnormalities are associated with a higher prevalence of subclinical damage in various organs, deranged renal function related to microalbuminuria, and increased risks of cardiovascular events and mortality [3, 4]. Detection of target organ damage plays a key role in the management of human patients with systemic hypertension and evaluation of their overall risk of mortality [3].

Microalbuminuria is defined as a urinary albumin excretion rate that is higher than normal, but lower than 200 *μ*g/min (the lowest detection limit of proteinuria as measured by standard laboratory methods), in the absence of urinary tract infection and acute illness, including myocardial infarction [5]. Microalbuminuria is a marker of general vascular dysfunction and target organ damage [6, 7]. In human patients, it serves as a predictor of higher risks of cardiovascular morbidity [6–8]. Isolated microalbuminuria has been associated with increased risks of cardiovascular events and mortality in humans [9].

The resistive index is calculated from blood flow velocities in vessels during the cardiac cycle, measured by pulsed-wave Doppler ultrasound [10]. An increased value of the resistive index (RI = [peak systolic velocity − end diastolic velocity]/peak systolic velocity) or the pulsatility index (PI = [peak systolic velocity − end diastolic velocity]/time averaged velocity) measured at the level of the interlobar arteries is associated with increased severity and duration of hypertension [11, 12]. Intrarenal RI is positively correlated with systemic blood pressure (SBP) and microalbuminuria, a sign of early target organ damage, in human patients [10, 13]. Intrarenal arterial RI is an independent predictor of microalbuminuria [13, 14]. These ultrasound Doppler-derived indices of renal vasculature reflect changes in intrarenal perfusion but are also associated with systemic hemodynamics and may provide useful prognostic information in human patients [15].

Systemic hypertension and proteinuria are commonly observed in dogs with HAC [16–19]. Glucocorticoid treatment can induce increased urine albumin excretion and glomerulosclerosis in dogs [20]. Mean values of renal RI and PI in dogs with HAC were significantly higher than those of healthy control dogs, suggesting that dogs with HAC experienced higher intrarenal vascular resistance [21]. We hypothesized that dogs with HAC would have a higher prevalence of systemic hypertension and subsequent target organ damage, with increased intrarenal RI values and microalbuminuria. Therefore, the objective of the present study was to evaluate associations between SBP, renal RI and PI, and microalbuminuria in dogs with HAC. Predictors of overall mortality associated with renal damage were also investigated.

## 2. Materials and Methods

### 2.1. Animals

Medical records of dogs that were examined at the Small Animal Internal Medicine Division of the National Taiwan University Veterinary Hospital between June 2009 and March 2015 and that met the following criteria were reviewed retrospectively: clinical signs consistent with HAC, results of routine serum biochemical and adrenocorticotropic hormone (ACTH) stimulation tests consistent with HAC, and performance of abdominal and adrenal ultrasonographic examinations. Matched controls were selected from a population of apparently healthy client-owned dogs that visited the National Taiwan University Veterinary Hospital for routine wellness checkups. No control dog had any clinical complaint or abnormality on urinalysis, complete blood count, serum biochemical panel, ACTH stimulation test, or adrenal ultrasonographic examination. Cases were matched with controls by age (< 1 year), sex, and body weight (BW). All clients gave their informed consent, and the study was performed in accordance with guidelines of the Research Ethics Office of the National Taiwan University Veterinary Hospital.

### 2.2. Review of Medical Records

The following data were extracted from medical records: baseline BW, results of SBP measurement, routine serum biochemical panel analyses, results of ACTH stimulation tests, urine albumin : creatinine ratio (UACR), abdominal ultrasonography, and measurement of renal vascular resistance. Survival time was calculated from the month of pituitary-dependent hyperadrenocorticism (PDH) diagnosis to the day of death or the end of the study period. All-cause mortality was the study endpoint. Treatments for PDH were also recorded.

### 2.3. Inclusion and Exclusion Criteria

Inclusion criteria were evidence of clinical signs (e.g., polydipsia, polyuria, polyphagia, decreased activity, panting, potbellied appearance, and dermatologic problems), results of routine serum biochemical analyses (i.e., elevated activities of hepatic enzymes), results of ACTH stimulation tests (i.e., post-ACTH cortisol concentration > 20 *μ*g/dL), and abdominal ultrasonographic evaluations that were consistent with a diagnosis of PDH [22]. Dogs with inconclusive ACTH stimulation test results were excluded. Normal or mildly and bilaterally enlarged adrenal glands were classified as PDH. Ultrasonographic findings of asymmetric, abnormally enlarged, irregularly shaped, or round adrenal glands, and findings of nodules/masses in the entire adrenal gland or in one pole with heterogenicity and mineralized foci, were classified as adrenal-dependent HAC [23, 24]. Dogs classified as adrenal-dependent HAC were excluded from the study. Dogs diagnosed with urinary tract infection, renal failure, hydronephrosis, or uro/nephrolithiasis and those treated with potentially nephrotoxic drugs were excluded from the study.

### 2.4. SBP Measurement

SBP values of healthy control dogs and dogs with PDH were measured by Doppler sphygmomanometry. For dogs with PDH, SBP was measured before commencement of treatment. A Doppler ultrasonic flow detector with an inflatable cuff was used. The cuff that was approximately 40% of the antebrachial circumference was selected for each dog and wrapped around the middle part of the antebrachium. A Doppler probe coated with ultrasonic transmission gel was positioned over the palmar area to detect blood flow from the arteria digitalis palmaris communis. The cuff was inflated and deflated to obtain the SBP reading via an aneroid pressure gauge. During SBP measurement, the antebrachium was maintained at the level of the heart. A series of five readings (with 10 to 20 seconds between consecutive measurements) was obtained for each dog. To minimize procedural stress, all dogs were allowed to assume a comfortable position with only gentle restraint by their owners. Dogs remained in the same position throughout SBP measurement. The final SBP value was calculated as the mean of five readings. Systemic hypertension was defined as SBP ≥ 160 mmHg [25].

### 2.5. Microalbuminuria Assay and UACR

Freshly voided urine samples were collected from all dogs in both groups. For dogs with PDH, the urine sample was collected before commencement of treatment. Urine albumin analysis was carried out within four hours of urine collection. Sediments in the urine samples were checked by light microscopic examination. Samples with hematuria (≥ 10 red blood cells per high-power field), pyuria (≥ 5 white blood cells per high-power field), or bacteriuria were excluded from UACR analysis and the study. Urine albumin and creatinine concentrations were determined by semiquantitative analysis, and UACR was calculated accordingly. In this study, no microalbuminuria was defined as UACR < 0.03, microalbuminuria as UACR = 0.03–0.3, and albuminuria as UACR > 0.3 [19].

### 2.6. Vascular Resistance Measurements

Triplex Doppler ultrasonography and a high-resolution multifrequency convex probe (4–8 MHz) were used to obtain the RI and PI values of the aortic flow velocity. During this process, the dog was not sedated and was in the right lateral recumbent position. The aortic RI and PI were measured at the level of the left kidney. Final RI and PI values of the aorta were calculated as the means of six measurements of the aortic flow velocity by using spectral Doppler waveforms.

To obtain the RI and PI values of renal blood flow, color Doppler was used to visualize the intrarenal vasculature. Pulsed Doppler and a high-resolution multifrequency convex probe (same as above) were used to detect renal blood flow in one interlobar or arcuate artery. During this procedure, the nonsedated dog was placed in the right or left lateral recumbent position. The RI and PI values for each kidney were calculated as the means of nine measurements of vascular flow velocities by using spectral Doppler waveforms in the arteries at three different locations [20].

Intraobserver coefficients of variation for RI and PI values were calculated through a variance component analysis. Six clinically healthy dogs were scanned on three nonconsecutive sessions throughout the same day by a single examiner (HYC). The same single examiner (HYC) performed all aortic and renal RI and PI measurements and was blinded to the initial examination results.

### 2.7. Statistical Analysis

Continuous variables are summarized as mean ± standard deviation (SD). The D'Agostino-Pearson omnibus and Shapiro-Wilk tests were used to test the normality of all data distributions. All tests were two-tailed. A *P* value < 0.05 was considered statistically significant. All continuous data were normally distributed.

Overall group-wise continuous data (BW, SBP, baseline serum albumin, and creatinine concentrations, results of ACTH stimulation tests) were evaluated by one-way analysis of variance to compare the results between the control and PDH groups. Left and right renal RI and PI values in each group were compared by paired *t*-tests. Multivariate general linear model analyses were performed for microalbuminuria status (no microalbuminuria and microalbuminuria versus albuminuria), renal vascular resistance status (normal versus increased renal RI/PI), and health status (control versus PDH) as outcomes and predictors. SBP status (hypertensive [SBP ≥ 160 mmHg] versus normotensive) was included as a covariate. Further discriminant analysis was carried out to provide a predictive model for microalbuminuria status from independent variables (health status, sex, SBP status, and mean renal RI and PI). Similar models were applied to identify predictive markers for increased renal RI based on health status, sex, and SBP.

Kaplan-Meier curves and log-rank tests were used to compare potential prognostic variables for survival time and mortality of PDH. These variables included baseline SBP (hypertensive [SBP ≥ 160 mmHg] versus normotensive), baseline renal RI (increased [RI ≥ 0.70] versus normal), baseline renal PI (increased [RI ≥ 1.45] versus normal), and baseline UACR (albuminuria and microalbuminuria versus no microalbuminuria). Dogs that were alive at the time of data analyses were censored in the survival analysis, and the last known date alive was used for dogs lost to follow-up. Variables with *P* < 0.10 in the univariate analysis were included in a forward stepwise manner to build the multivariate Cox proportional-hazard regression model to assess the associations between risk of death and the explanatory variables. Risk was quantified by hazard ratios (HRs) with 95% confidence intervals (CIs).

## 3. Results 

### 3.1. Baseline Characteristics

In total, 20 PDH dogs and 20 matched-control dogs were included in this study. Breeds of the 20 PDH dogs were Maltese Terrier (*n* = 7), mixed breed (6), Miniature Schnauzer (2), Yorkshire Terrier (2), Miniature Dachshund (1), Pomeranian (1), and Shih Tzu (1). Breeds of 20 matched-control dogs were Maltese Terrier (*n* = 7), mixed breed (4), Pomeranian (3), Shih Tzu (2), Yorkshire Terrier (2), Miniature Dachshund (1), and Miniature Schnauzer (1). Baseline characteristics of both groups are shown in Table 1. Serum creatinine concentrations were not significantly different between PDH dogs (0.8 ± 0.4 mg/dL) and controls (0.9 ± 0.3 mg/dL). The mean SBP was higher in the PDH group (148.8 ± 13.9 mmHg) than in the control group (132.8 ± 12.1 mmHg; *P* = 0.001; Table 1). Incidence rates of systemic hypertension (SBP ≥ 160 mmHg) in the control and PDH groups were 5% and 35% (*P* = 0.001), respectively.

### 3.2. UACR

Among the 20 controls, 75% showed neither microalbuminuria nor albuminuria, and 25% had microalbuminuria. Among the 20 dogs with PDH, 30% showed no microalbuminuria, 30% had microalbuminuria, and 40% had albuminuria (Table 1). The prevalence of albuminuria was higher in the PDH group than in the control group (*P* < 0.001).

### 3.3. Aortic/Renal RI and PI

Aortic RI and PI values in the control group were 0.92 ± 0.04 and 3.13 ± 0.64, respectively. Aortic RI and PI values in the PDH group were 0.91 ± 0.07 and 2.87 ± 0.50, respectively. No difference in aortic RI (*P* = 0.496) or PI (*P* = 0.089) was detected between the two groups (Table 2). Renal RI and PI values derived from the right and left kidneys did not differ significantly (*P* = 0.103 and *P* = 0.175, resp.).

Comparisons of renal RI and PI values between groups were expressed as the mean values of the right and left kidneys. The mean renal RI and PI values were significantly higher in the PDH group (0.69 ± 0.06 and 1.39 ± 0.50, resp.) than in the control group (0.65 ± 0.03 and 1.20 ± 0.12, resp.; both *P* < 0.001; Table 2).

The upper cutoff values of the reference ranges of renal RI and PI values (0.70 and 1.45, resp.) were calculated as mean ± 2 SDs of the indices of the clinically healthy controls. Based on these cutoff values, the incidence of increased renal RI (≥ 0.70) was 50% in the PDH group and 5% in the control group (*P* < 0.001), whereas the incidence of increased renal PI (≥ 1.45) was 35% in the PDH group and 0% in the control group (*P* < 0.001; Table 2). Coefficients of variation for the renal RI and PI and aortic RI and PI values in the six healthy dogs were 2.8%, 4.5%, 7.1%, and 9.1%, respectively.

### 3.4. Associations among SBP, Renal RI/PI, and Albuminuria

The association between albuminuria and PDH was significant (*P* = 0.008). Presence of albuminuria was not affected by sex, SBP status, or renal RI or PI status. Results of the discriminant analysis indicated that PDH was the absolute coefficient in this predictive model for albuminuria (*P* = 0.002). Sex, SBP status, and renal RI and PI status did not contribute to the predictive model. PDH was the only predictor of increased renal RI (*P* = 0.007) or PI (*P* = 0.035).

### 3.5. Survival Analyses

**Figure 1:**
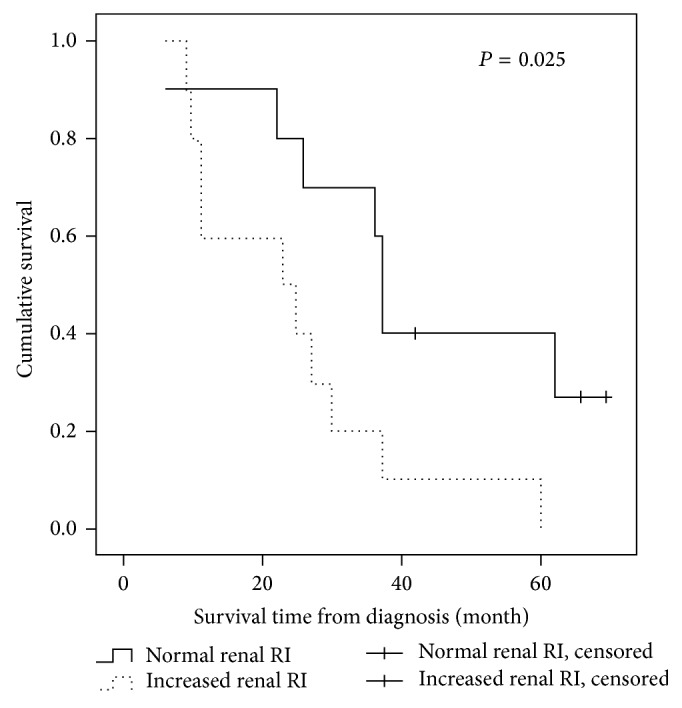
Kaplan-Meier curves indicating the survival probability after diagnosis of hyperadrenocorticism in 20 dogs, categorized by the presentation of an increased renal resistive index (≥ 0.70). Data for dogs that were alive at the conclusion of the study (*n* = 3) were censored (indicated by hatch marks).

Among the 20 dogs with HAC, 19 dogs were treated with trilostane (1.5–2.5 mg/kg,* q* 12 hours) and one dog was treated with ketoconazole (12 mg/kg,* q* 12 hours) for the condition of PDH. During the observation period (from June 2009 to March 2015), 17 (85.0%) HAC dogs died or were euthanized. Causes of death were advanced cardiac diseases (*n* = 10), advanced neurological disorders (*n* = 4), diabetes mellitus (*n* = 2), and multiple organ neoplasia (*n* = 1). No deaths were attributed to renal failure. The mean survival time was 32.4 ± 19.7 (range, 6–70) months. Survival time was related significantly to renal RI status (*P* = 0.025; Figure 1). Survival time was shorter in dogs with mean renal RI ≥ 0.70 (median 23 months, 95% CI 1.3–44.7 months) compared to dogs with normal renal RI values (median 37 months, 95% CI 35.5–38.5 months). Survival was not affected by systemic hypertension, increased mean renal PI, or albuminuria.

Of the four variables used as predictors in the univariate analysis, increased mean renal RI (HR 6.1, 95% CI 0.9–40.5, and *P* = 0.044) was associated with survival. Increased mean renal PI (HR 1.2, 95% CI 0.4–3.6, and *P* = 0.063), albuminuria (HR 0.8, 95% CI 0.2–3.9, *P* = 0.235), and systemic hypertension (HR 0.4, 95% CI 0.1–1.7, and *P* = 0.809) were not predictors of poor outcomes. No interaction was detected among variables remaining in the final multivariate model. Increased mean renal RI (HR 16.2, 95% CI 2.4–111.3, and *P* = 0.005) was the only predictor associated with the outcome.

## 4. Discussion 

In this study, the mean SBP, mean renal RI and PI, and the incidence of systemic hypertension, albuminuria, and increased renal RI and PI were significantly higher in the PDH group than in the control group. These results are consistent with findings from previous studies, in which systemic hypertension, increased renal RI, and albuminuria were common in dogs with PDH [19, 26].

Although a correlation between systemic hypertension, increased renal RI, and albuminuria was expected, increased renal RI was not associated with systemic hypertension in this study. Additionally, albuminuria was not associated with systemic hypertension and increased renal RI or PI. PDH was the only predictor of increased renal RI and albuminuria. These findings are not consistent with studies of human patients, which reported a positive correlation of renal RI with SBP and an association of increased renal RI with microalbuminuria [10, 13, 14].

In previous studies, dogs with HAC exhibited significantly higher renal RI values [20], but these values were not correlated with the SBP measurements [27]. Our results do not support a correlation between systemic hypertension, increased renal RI, and albuminuria in dogs with PDH. Furthermore, we did not find any correlation between SBP and renal RI in dogs with PDH and renal complications. Based on the findings of this study, renal RI is not an ideal marker for target organ damage secondary to systemic hypertension in dogs with PDH.

The RI is considered to be an indicator of renal vascular resistance in humans [11]. Several hemodynamic and physiological factors influence intrarenal arterial Doppler waveform patterns and, consequently, the RI value. These factors include vascular compliance (arterial stiffness), pulse pressure, interstitial pressure, urethral pressure, intra-abdominal pressure, and plasma renin activity [28–33]. Increased renal RI was reported in human patients and dogs with hepatic complications [33, 34]. We excluded dogs diagnosed with renal failure, hydronephrosis, or nephrolithiasis and those treated with potentially nephrotoxic drugs from the present study. Therefore, the impact of PDH on renal RI could also be attributed to factors other than intrarenal factors, such as steroid hepatopathy and increased plasma renin activity, which was reported in dogs with PDH [35].

An increased renal RI value was found to be a marker for glomerulosclerosis and a predictor of global outcome in critically ill human patients [36, 37]. Renal RI is associated independently with cardiovascular, systemic vascular, and chronic kidney diseases in human patients [37]. Glomerulosclerosis can be induced by glucocorticoid treatment in dogs [38]. We were unable to clarify the correlation between glomerulosclerosis and increased renal RI. However, 17 of the 20 dogs with PDH died or were euthanized, and no deaths were attributed to renal failure.

In this study, increased renal RI was the only predictor of survival. Survival was not affected by systemic hypertension, increased renal PI, or albuminuria. Prognoses were less favorable for dogs with increased renal RI than for dogs with normal renal RI values. The deranged renal function associated with PDH did not significantly affect the survival of these dogs. The increased renal RI might reflect changes in systemic hemodynamics, such as increased plasma renin activity in dogs with PDH [35]. Increased renal vascular resistance was correlated with increased plasma renin activity [33]. Furthermore, the activated renin-angiotensin-aldosterone system plays an important role in endothelial dysfunction and myocardial remodeling in the progress of heart failure [39, 40]. Advanced cardiac disease was the major cause of this cohort. This was also consistent with the result of our previous study [22]. These might explain the link between increased renal RI and poor outcome in dogs with PDH. Further study to elucidate the pathophysiological association between increased renin activity, increased renal RI, and cardiac disorders is warranted in dogs with PDH. Based upon the finding of this study, a renal RI value ≥ 0.7 was a strong independent predictor of all-cause mortality in dogs with PDH. This finding indicates that renal RI could be used as a predictor for overall outcome in dogs with PDH. Thus, we recommend the evaluation of renal RI in the assessment and further management of dogs with PDH.

Renal RI is used as an indicator of renal vascular resistance in many studies. However renal PI may be more accurate in differentiating abnormal wave forms because it also incorporates mean velocity was a parameter [33]. Renal PI has been reported to be positively correlated with levels of bilirubin in human patients with hepatic cirrhosis and levels of creatinine in dogs with renal diseases [41] compared to renal RI. In this study, the mean renal PI and the incidence of increased renal PI were significantly higher in dogs with PDH compared to the controls, but it did not affect survival in these dogs. It has been reported that survival time was not affected by serum alkaline phosphatase and alanine aminotransferase activities in dogs with PDH [22]. Furthermore, no death was attributed to advanced hepatic nor renal disorders in this study. Survival was not affected by the presence of steroid hepatopathy or deranged renal function in dogs with PDH. These might explain the lack of an association between increased renal PI and survival in this study.

The incidence of microalbuminuria was not different between the PDH and control groups. However, a higher incidence of albuminuria was found in dogs with PDH compared to controls. Furthermore, there was a significant association between albuminuria and PDH, with PDH being the only predictor of albuminuria. Nevertheless, the correlation among SBP, renal RI, and albuminuria was not supported in this study.

Apart from hypertension, many complications associated with HAC in humans are also linked with an increased urinary albumin excretion rate. Several mechanisms, such as elevated plasma glucose levels and abnormalities in lipid metabolism, contribute to the increased leakage of albumin into the urine of human patients with HAC [42, 43]. These complications are also seen commonly in dogs with HAC [44]. These mechanisms might explain the lack of an association between albuminuria and SBP in these dogs. The pathophysiological cause of albuminuria in dogs with PDH was not clarified in this study. Nevertheless, the presence of albuminuria did not affect survival significantly in dogs with PDH.

Several limitations of this study must be mentioned. The low-dose dexamethasone suppression test is the screening test of choice for HAC in dogs, unless iatrogenic HAC is suspected. In the area where the authors practice, the iatrogenic forms of HAC are more common than those of spontaneous origin. Therefore, an ACTH stimulation test was used for screening to determine the function of the adrenal glands. The association between survival and causes of death was not assessed. Additionally, the severity of glomerulosclerosis was not assessed in the deceased cases, and the association of increased renal RI and deranged renal function was not clarified. Finally, the study was limited by the lack of comparison of survival times with those derived from controls without PDH.

## 5. Conclusions

The incidence rates of systemic hypertension, increased renal RI and PI, and albuminuria, and the mean values of renal RI and PI were significantly higher in the PDH group than in controls. No correlation among systemic hypertension, increased renal RI, and albuminuria was found. The presence of PDH was the sole predictor of increased renal RI and albuminuria. Survival was not affected by increased renal PI, systemic hypertension, or albuminuria. Increased renal RI (≥ 0.70) was the only predictor of overall mortality in dogs with PDH.

## Figures and Tables

**Table 1:** Baseline characteristics of dogs.

Variable	Control (*n* = 20)	PDH (*n* = 20)
Age (years)	11.8 ± 2.9	12.2 ± 2.5
Body weight (kg)	6.3 ± 3.5	7.0 ± 3.8
Sex (female : male)	16 : 4	17 : 3
Heart rate (bpm)	121.2 ± 13.9	127.1 ± 20.3
Indirect SBP (mmHg)	132.8 ± 12.1	148.8 ± 13.9^*∗*^
Prevalence of hypertension (≥ 160 mmHg)	5% (1/20)	35% (7/20)^*∗*^
Serum albumin (g/dL)	3.3 ± 3.0	3.4 ± 4.0
Serum creatinine (mg/dL)	0.9 ± 0.3	0.8 ± 0.4
Microalbuminuria (UACR 0.03–0.3)	25% (5/20)	30% (6/20)
Albuminuria (UACR > 0.3)	0% (0/20)	40% (8/20)^*∗*^
Pre-ACTH cortisol (*μ*g/dL)	1.8 ± 0.7	4.8 ± 3.0^*∗*^
Post-ACTH cortisol (*μ*g/dL)	10.3 ± 2.1	29.3 ± 8.0^*∗*^

Data are presented as mean ± SD or % (*n*). ACTH, adrenocorticotropic hormone; PDH, pituitary-dependent hyperadrenocorticism; SBP, systemic blood pressure; UACR, urine albumin to creatinine ratio.

^*∗*^ *P* < 0.05 relative to control.

**Table 2:** Resistive and pulsatility index of renal artery and aorta of dogs.

Variable	Control (*n* = 20)	PDH (*n* = 20)
Aorta RI	0.92 ± 0.04	0.91 ± 0.07
Aorta PI	3.13 ± 0.64	2.87 ± 0.50
Renal RI	0.65 ± 0.03	0.69 ± 0.06^*∗*^
Renal PI	1.20 ± 0.12	1.39 ± 0.27^*∗*^
Increased renal RI (%)	1/20 (5%)	10/20 (50%)^*∗*^
Increased renal PI (%)	0/20 (0%)	7/20 (35%)^*∗*^

Data are presented as mean ± SD or *n* (%). PDH, pituitary-dependent hyperadrenocorticism; PI, pulsatility index; RI, resistive index.

^*∗*^ *P* < 0.05 relative to control.
